# A Self-Powered Multifunctional Bracelet for Pulse Monitoring and Personal Rescue

**DOI:** 10.3390/bios13050552

**Published:** 2023-05-16

**Authors:** Wei Sun, Jiangtao Xue, Puchuan Tan, Bojing Shi, Yang Zou, Zhou Li

**Affiliations:** 1 Beijing Institute of Nanoenergy and Nanosystems, Chinese Academy of Sciences, Beijing 101400, China; sunwei@binn.cas.cn (W.S.); xuejiangtao@binn.cas.cn (J.X.); tanpuchuan@binn.cas.cn (P.T.); 2 School of Nanoscience and Engineering, University of Chinese Academy of Sciences, Beijing 100049, China; 3 School of Life Science, Institute of Engineering Medicine, Beijing Institute of Technology, Beijing 100081, China; 4 Key Laboratory for Biomechanics and Mechanobiology of Ministry of Education, Beijing Advanced Innovation Centre for Biomedical Engineering, School of Biological Science and Medical Engineering, School of Engineering Medicine, Beihang University, Beijing 100191, China; bjshi@buaa.edu.cn; 5 Center on Nanoenergy Research, School of Physical Science and Technology, Guangxi University, Nanning 530004, China

**Keywords:** self-powered, pulse monitoring, energy harvesting, nanogenerator, wearable

## Abstract

For outdoor workers or explorers who may be exposed to extreme or wild environments for a long time, wearable electronic devices with continuous health monitoring and personal rescue functions in emergencies could play an important role in protecting their lives. However, the limited battery capacity leads to a limited serving time, which cannot ensure normal operation anywhere and at any time. In this work, a self-powered multifunctional bracelet is proposed by integrating a hybrid energy supply module and a coupled pulse monitoring sensor with the inherent structure of the watch. The hybrid energy supply module can harvest rotational kinetic energy and elastic potential energy from the watch strap swinging simultaneously, generating a voltage of 69 V and a current of 87 mA. Meanwhile, with a statically indeterminate structure design and the coupling of triboelectric and piezoelectric nanogenerators, the bracelet enables stable pulse signal monitoring during movement with a strong anti-interference ability. With the assistance of functional electronic components, the pulse signal and position information of the wearer can be transmitted wirelessly in real-time, and the rescue light and illuminating light can be driven directly by flipping the watch strap slightly. The universal compact design, efficient energy conversion, and stable physiological monitoring demonstrate the wide application prospects of the self-powered multifunctional bracelet.

## 1. Introduction

Outdoor workers or explorers usually remain in extreme environments for a long time and may face emergencies such as being trapped, lost, or in danger [1]. Some wearable electronic devices integrated with health monitoring and emergency functions, such as smart watches, may protect their lives [2,3,4]. However, these devices are typically powered by batteries, leading to a limited service time [5,6,7]. Especially in emergencies, the lack of power in electronic devices can make people feel desperate [8]. Therefore, the development of low-power physiological sensors and emergency energy supply systems is of great significance for long-term health monitoring and personal rescue of outdoor workers or explorers [9,10].

Compared with solar energy [11,12,13], wind energy [14,15,16,17], or thermal energy [18,19,20], the mechanical energy from the human body can be used as an effective energy source anytime and anywhere without being limited by the environment and weather [21]. An electromagnetic generator (EMG) has been used up to now due to its high conversion efficiency and reliability [22], but it also has some limitations such as a large volume and weight, and low output voltage under low frequency conditions [23]. As emerging energy harvesting technologies, triboelectric nanogenerators (TENGs) and piezoelectric nanogenerators (PENGs) are capable of converting mechanical energy from the human body or the environment into electrical energy and exhibit high applicability over a wide frequency range [24,25,26,27]. A TENG generates electricity based on the coupling effect of contact electrification and electrostatic induction [28,29,30], while a PENG produces electricity by the piezoelectric effect [31,32]. Due to their good flexibility, lightweight, low cost, and high voltage output characteristics, TENGs and PENGs have attracted much attention in recent years [33,34,35]. In addition, TENGs and PENGs can directly convert tiny mechanical signals into electrical signals, serving as self-powered sensors to detect the physiological signals of the human body [36]. Since no external power source is required, the self-powered sensors based on TENGs or PENGs enable long-term sensing and monitoring in a variety of scenarios [37,38].

Currently, many versatile smart wearable devices are commercially available but, except for a small number of devices with a solar charging function, few products can perform self-charging in extreme environments and emergencies [39]. In addition, the stable monitoring of physiological signals during exercise is also a challenge [40]. In this study, we developed a self-powered multifunctional bracelet (SPMB), which integrated a hybrid energy supply module (HESM) based on EMGs and PENGs in the inherent structure of the watch, as well as a coupled pulse monitoring sensor (CPMS) based on TENGs and PENGs. The HESM is designed to simultaneously harvest the rotational kinetic energy and elastic potential energy of the watch strap when it is swung. The power generated by HESM can charge a commercial lithium-ion battery or directly drive some low-power functional electronic components. In adopting a statically indeterminate structure design and reducing pre-tension, the CPMS can stably monitor the pulse physiological signals of the wearer during movement, with strong anti-interference ability. The functions of the SPMB in real-time pulse signal transmission, in Global Position System (GPS) positioning, in the driving of rescue lights, and in lighting were further verified, indicating the potential of the SPMB for vital sign monitoring and personal rescue in emergencies for outdoor workers and explorers. In addition, the compact structural design of the SPMB does not increase the size of the watch itself, and is generally compatible with common mechanical and electronic watches on the market.

## 2. Materials and Methods

### 2.1. Fabrication of DC-EMG Module of HESM

Molds for the supporting the structure of the direct-current electromagnetic generator (DC-EMG) module were printed using a three-dimensional printer (Raize 3D E2, Shanghai, China) and polylactic acid (PLA, Raise 3D, Shanghai, China) printing supplies. The thickness of each layer of deposited PLA was 0.15 mm. Reverse drive brushed the direct current motor (ZWPD06-136, Shenzhen, China) as an electromagnetic generator. The overrunning clutch (HF0306, Nuremberg, Germany) had an inner diameter of 3 mm, an outer diameter of 6.5 mm, and a thickness of 6 mm. The EMG frame and strap, as well as the outer ring of the overrunning clutch and the watch case, were fixed with 422 glue.

### 2.2. Fabrication of PENG Module of HESM

A commercially available polyvinylidene fluoride (PVDF, ZHIMK Technology Company, Shenzhen, China) material with silver coating on both sides was used as the piezoelectric material. The thickness of PVDF before silver plating was 28 μm, and the total thickness of silver plating layers on both sides was 12 μm. The PVDF material was 6 cm long and 1.5 cm wide. The PVDF was placed in a cavity of a 3D-printed mold (6.1 cm long, 1.6 cm wide) made of PLA material, and then 3 mL of polydimethylsiloxane (PDMS, Sy-gard 184, East Lansing, MI, USA) was poured into the mold to encapsulate the PVDF. The encapsulated PVDF was taken after curing in a 60 °C drying oven for 6 h. Ultimately, the encapsulated PVDF was fixed to the strap using 422 glue.

### 2.3. Fabrication of CPMS

Initially, a 0.13 mm thick polytetrafluoroethylene (PTFE) tape (Wuxi Shunxuan New Materials Company, Wuxi, China) was adhered to a 0.1 mm thick titanium sheet (Wenghou Metal Materials Company, Hefei, China) to serve as the upper structural layer. The titanium sheet was 3 cm long and 1 cm wide. The PTFE tape was slightly larger than the size of the titanium sheet, allowing it to cover the titanium sheet and prevent short circuits between the exposed titanium sheet and subsequent lower structural layers. The lower structural layer is comprised of commercial PVDF with silver plating on both sides, which was 3 cm long and 1 cm wide. The PVDF was 28 μm thick before silver plating, and the total thickness of silver plating layers on both sides was 12 μm. Subsequently, the lower structural layer was bonded to the lower encapsulation layer’s PTFE tape. Next, four pieces of 0.8 mm thick very high bonding (VHB) double-sided tape (3M 4910, 3M Company, Saint Paul, MN, USA) were affixed on the lower structure layer and 1 mm away from the other two sides of the lower structural layer. Then, the upper structural layer was bonded with VHB tape to align it with the lower structural layer. After that, the device was encapsulated with PTFE tape, serving as the upper encapsulation layer. Excluding the structural layer and supporting layer and their edges, the redundant encapsulation layer was trimmed. The thicknesses of the encapsulation layers were 0.13 mm. The encapsulated CPMS was 3.6 cm long, 2.1 cm wide, and 1.2 mm thick. The length and width dimensions of the encapsulated CPMS depended on the size of the encapsulation layer. Subsequently, 1 mL of liquid silicone elastomer (Sil-Poxy, Smooth-On, Macungie, PA, USA) was applied to the gap between the CPMS and the watch strap. The silicone elastomer was filled in the gap of the CPMS and watch strap by slowly flowing on its own by slight pressing. The silicone elastomer became solid after curing at room temperature for 20 min to complete the fitting of CPMS and the tape. The thickness of the silicone elastomer was about 0.5 mm at its maximum.

### 2.4. Functional Electronic Component

For the electromagnetic boost, a commercial booster module was used. Commercial GPS positioning systems were used to obtain longitude and latitude information. For wireless data transmission, commercial Bluetooth was used. Molds for the watch case were printed using a three-dimensional printer (Raize 3D E2, Shanghai, China) and PLA printing supplies. A commercial lithium-ion battery (Tengfei New Energy Company, Shenzhen, China) of 30 mAh was used to store electrical energy. The lithium-ion battery was 9 mm long, 8.5 mm wide, and 4.5 mm thick. The dimensions of the interior space of the watch case housing the functional electronic component were 5.1 cm long, 2.3 cm wide, and 1.6 cm thick.

### 2.5. Characterization

A Servo motor (80ST-G02430, Wenzhou, China) and a programmable controller (TC5510, Beijing, China) were used to provide angular rotation for the HESM to maintain periodic motion. The current lower than 1 mA and the transferred charge were measured by an electrometer (Keithley 6517, Tektronix, Beaverton, OR, USA). The current higher than 1 mA was calculated by measuring the voltage with a high-impedance probe (HP9258, PINTECH, Taiwan, China) and connecting a fixed resistance in parallel. All voltage data were measured by a high-impedance probe. The electrical data were recorded using an oscilloscope (Teledyne LeCroy HDO 8108A, Teledyne LeCroy, New York, NY, USA).

## 3. Results and Discussion

### 3.1. Overview of Self-Powered Multifunctional Bracelet

**Figure 1:**
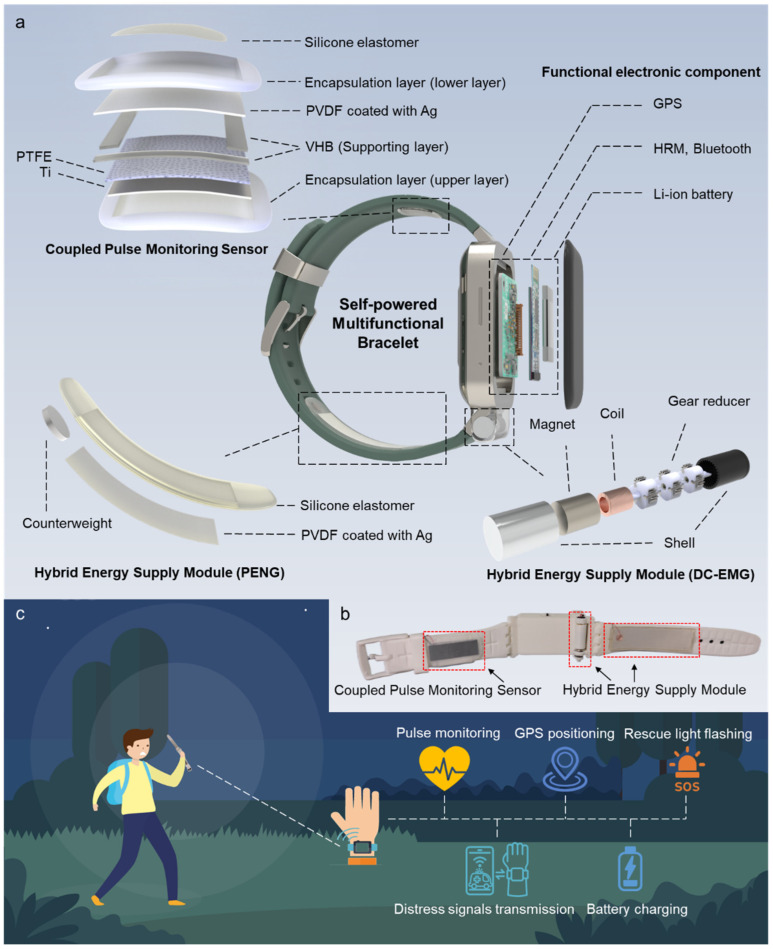
Structure and application of SPMB. (**a**) Schematic diagram and exploded view of SPMB. (**b**) Photo of SPMB. (**c**) Schematic illustration showing the practical application of SPMB.

The self-powered multifunctional bracelet consists of two parts: the watch case and the watch strap. The watch strap includes a HESM composed of a EMG and PENG, and a CPMS based on the coupling of a TENG and PENG, while the watch case contains functional electronic components (Figure 1a).

A direct-current electromagnetic generator (DC-EMG) of HESM and its auxiliary structures are located at the connection between the watch case and the watch strap. The DC-EMG module consists of a brushed electromagnetic generator and a three-stage gear reducer. In conjunction with the structural design of the connection between the watch case and the watch strap, the DC-EMG generates direct current during the periodic opening and closing motion of the two components. The direct current principle of DC-EMG will be discussed in detail in the following section. The PENG module in the HESM is located in the watch strap on the same side as the EMG module, 3 cm away from the connection between the watch strap and the watch case. During the swinging process of the watch strap, both the DC-EMG and PENG modules are driven to move together, serving as HESM. The photo of the SPMB is shown in Figure 1b.

The CPMS is attached to the inner side of the watch strap, close to the pulse. The CPMS is composed of an encapsulation layer (upper layer), upper structural layer, support layer, lower structural layer, encapsulation layer (lower layer), and connecting layer. The upper and lower encapsulation layers are made of polytetrafluoroethylene (PTFE), the upper structural layer consists of a titanium sheet and PTFE, the lower structural layer is PVDF coated with Ag on both sides, and the connecting layer is made of silicone elastomer.

The SPMB can achieve pulse monitoring, GPS positioning, rescue light flashing, wireless signal transmission, and battery charging (Figure 1c). CPMS collects pulse wave signals and converts them into electrical signals, which are processed by a heart rate monitoring module (HRM) and transmitted to the smartwatch or mobile phone via Bluetooth, realizing real-time monitoring of vital signs. The HESM is used to drive the GPS positioning system, Bluetooth module, and HRM. Meanwhile, the HESM can activate the distress signal light and charge the lithium-ion battery, catering to the long-term power and basic physiological signal monitoring requirements of outdoor workers or adventurers. In addition, due to the compact structural design, SPMB can easily be combined with commercial smart watches. In the future, through integration, when the wearer’s vital signs change, such as the wearer is in distress or in a dangerous state, it is possible to achieve the function of automatic distress signal transmission.

### 3.2. Principles and Characterization of DC-EMG and PENG Module of HESM

**Figure 2:**
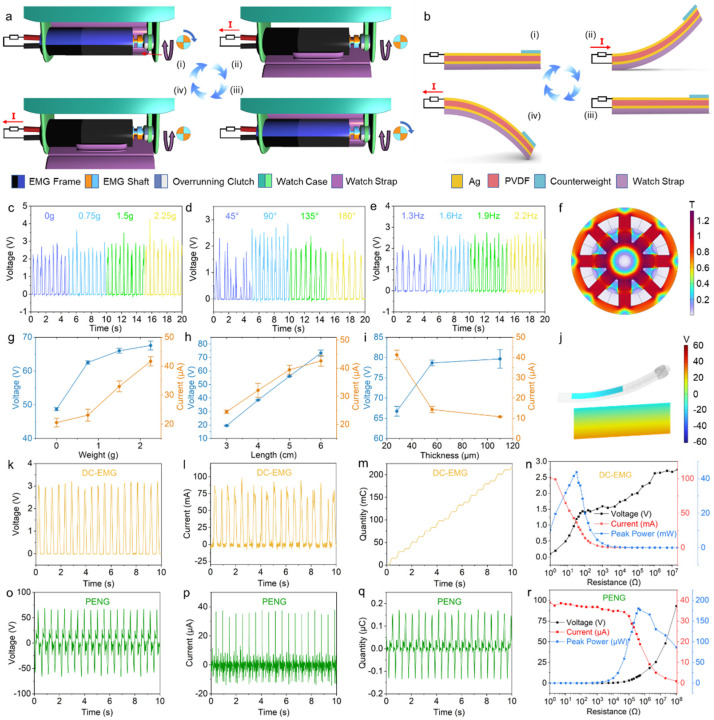
Principles and characterization DC-EMG module and PENG module. (**a**) Working principle of DC-EMG of HESM. (**b**) Working principle of PENG of HESM (**c**–**e**) Effects of counterweight mass, rotation angle, and frequency on DC-EMG. (**f**) Simulation results of magnetic flux density on DC-EMG. (**g**–**i**) Effects of counterweight mass, length, and thickness on the voltage and current output of PENG. (**j**) Simulation result of electric field on PENG. (**k**–**m**) Voc, Isc, Qsc of DC-EMG. (**n**) Output voltage, current and peak power under different load resistors of DC-EMG. (**o**–**q**) Voc, Isc, Qsc of DC-EMG. (**r**) Output voltage, current, and peak power under different load resistors of PENG.

The EMG shell of the DC-EMG module is fixed to the edge of the watch case, while the torque shaft of the generator is rigidly connected to the inner ring of an overrunning clutch, which is in turn fixed to the outer shell of the watch (Figure 2a). After fixing the outer ring of the overrunning clutch, the inner ring can rotate with low resistance in one direction while self-locking in the other direction (Figure S1). In one of the directions of rotation, the inner and outer rings of the overrunning clutch rely on the rollers to engage each other to transmit torque. Since the outer ring and the watch case are fixed to each other, a self-locking effect is achieved. In another direction of rotation, the rollers between the inner ring and the outer ring are forced to compress the spring inside the overrunning clutch, so that the inner ring and the outer ring are separated from each other to realize the separation of motion.

When the watch strap is rotated 90° relative to the outer end of the watch case (Figure 2a (ii)), the frame of the EMG rotates with the watch strap, while the EMG shaft remains stationary due to the tendency of the overrunning clutch to self-lock in this direction, causing the EMG frame to rotate relative to the EMG shaft for one-quarter cycle and generating an electric current. When the watch strap is rotated 90° in the opposite direction, as shown in Figure 2a (iii), the EMG frame rotates in the opposite direction by 90°, while the shaft can rotate with minimal resistance in this direction due to the overrunning clutch, causing no relative rotation between the frame and shaft, and thus no electrical output from the generator. This process repeats itself in the next cycle of movement, as the watch strap rotates in the positive direction to reach the position of Figure 2a (iv), generating an electrical current in the same direction as in Figure 2a (ii). When the watch strap continues to rotate in the opposite direction, it returns to state (i). Thus, when the watch strap is shaken relative to the watch case, only a unidirectional electrical output is produced.

There are two reasons why we decided to introduce an overrunning clutch instead of directly fixing the shaft to the watch case. On the one hand, it avoids the introduction of a rectifier bridge, reduces the volume of the HESM, and can also reduce the decrease in the electromagnetic voltage, making the output voltage just enough to provide DC power to some electrical devices. In addition, reducing the wiring part of the rectifier bridge also enables the DC-EMG module to work normally under some harsh conditions, with higher robustness. On the other hand, when the strap swings at a high frequency, the gear reducer and EMG shaft can maintain unidirectional movement, avoiding damage to the internal multi-stage gear reducers caused by multiple direction changes. Moreover, after the introduction of an overrunning clutch, the minimum torque required for the strap to start swinging is also reduced, making it easy to swing.

The EMG tail frame is placed in the hole inside the watch case, but they are not directly connected. The inner hole of the watch case is equivalent to a radial bearing, and when combined with the watch case connected by the outer ring of the overrunning clutch, it can restrain the DC-EMG module from twisting in the other two axial directions, increasing the stability and durability of the EMG. After the combination of the EMG and the reducer, the diameter is only 6 mm, and the total length is 18.8 mm. By replacing the unused outer shell structure and hinge part of the watch with the DC-EMG module, the module’s compact design does not increase the volume of the watch itself.

The working principle of the PENG module of the HESM is shown in Figure 2b. When the watch strap is shaken, the PENG on the surface of the strap is bent and deformed, which creates a potential difference between the upper and lower surfaces of the PVDF. This potential difference generates an electric current under an external load. As a result, when the watch strap is shaken, the PVDF undergoes periodic bending in both directions, creating an alternating current.

The DC-EMG module of HESM was explored and optimized for tip mass, rotation angle, and frequency. It was found that adding a small counterweight at the end of the strap can increase the output voltage by PENG, and the highest output voltage was obtained when the counterweight was 2.25 g (Figure 2c). This is because increasing the weight lowers the natural frequency of the watch strap, making it more compatible with the normal swing frequency of the human hand. However, further increasing the weight has little effect on increasing the voltage output and strains the strength of the structure of the DC-EMG module. When the watch strap rotates at an angle of 90°, the voltage is at its highest (Figure 2d). This is because the initial angular acceleration of the swing of the strap is the same and, as the angle increases, the deflection of the PVDF will increase. The energy transferred to the strap is mostly converted into elastic potential energy rather than rotational kinetic energy. On the other hand, smaller angles of movement limit the maximum relative rotational speed. The normal frequency of the swing or flicking of a watch strap by the human body is between 1 and 2 Hz. We performed characterization near this frequency range (Figure 2e). We found that increasing the frequency beyond 1.9 Hz has a minor effect on the increase in voltage peak value, and too fast a swing of the watch strap consumes more energy from the human body. Therefore, we chose a frequency of 1.9 Hz for the remaining characterization, which is close to the normal frequency of the swing of a watch strap by hand. We performed electromagnetic field simulations on the DC-EMG module, and Figure 2f shows the magnetic flux density distribution of the coil inside the electromagnetic generator. The magnetic flux density change over a period is shown in Figure S2. By calculating the magnetic flux density change, we obtained the voltage curve corresponding to the actual EMG data (Figure S3).

The PENG module of HESM is also explored and optimized for the weight, length, and thickness of PVDF. Tests showed that a slightly higher weight contributed to an increase in the piezoelectric voltage and current, and therefore we optimized the weight of the counterweight to 2.25 g (Figure 2g). Moreover, we characterized the electrical output of PVDF materials with different lengths at the same width, and we found that both voltage and current can be greatly improved with an increase in PVDF length (Figure 2h). In addition, we performed voltage and current characterizations on PVDF with thicknesses of 28 μm, 56 μm, and 110 μm. We found that as film thickness decreases, the voltage decreases slightly, whereas the current can increase substantially. The current of a PENG with a thickness of 28 μm can reach 41.29 μA, while that of a PENG with a thickness of 56 μm is only 14.32 μA. For the energy supply module of a PENG’s wearable or portable electronic devices, the voltage output generated by a PENG is often large enough, while the current is often too small to drive numerous electrical devices. Therefore, we selected a length of 6 cm and thickness of 28 μm for subsequent characterization. By analyzing the motion of the PVDF material on the watch strap through simulation (Figure 2j), we obtained piezoelectric voltages that were consistent with the actual outputs. The output of the piezoelectric material during one cycle is shown in Figures S4 and S5.

After optimizing the DC-EMG and PENG modules in the HESM, we obtained data on the optimized open-circuit voltage, short-circuit current, and transferred charge, and characterized the impedance matching of the DC-EMG and PENG modules (Figure 2k–r). The voltage and current of the DC-EMG module were approximately 3.1 V and 86 mA, respectively, with a half-cycle average transferred charge of 10.5 mC. The DC-EMG module reached a peak power of 43.8 mW at a 33 Ω load. The voltage and current of the PENG module were approximately 67 V and 36 μA, respectively, with a half-cycle average transferred charge of 0.15 μC. The EMG module reached a peak power of 180 μW at a 400 kΩ load. Electromagnetic generators have low voltage, high current, and are suitable for low resistance load, while piezoelectric generators have high voltage, low current, and are suitable for high resistance load. In addition, we performed durability characterizations on the DC-EMG and PENG. When the mass of the counterweight is 2.25 g and the swing frequency of the strap is 1.9 Hz, the output of the DC-EMG and PENG have undergone minimal change after 50,000 cycles (Figures S6 and S7).

### 3.3. Characterization of HESM

Since both the DC-EMG and PENG modules can convert mechanical energy from the swinging motion of the watch strap into electrical energy, we combined the advantages of the two modules to drive devices with different threshold voltage and load power, thereby expanding the practical application range of a HESM.

**Figure 3:**
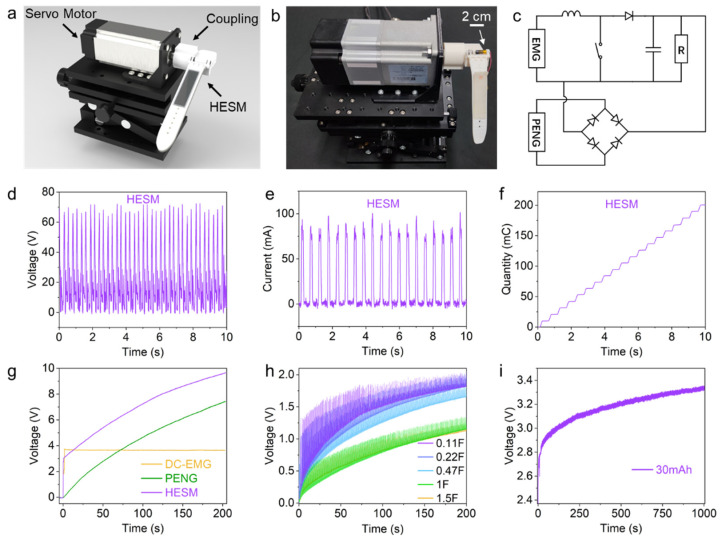
Characterization of HESM. (**a**) Schematic diagram of characterization test of HESM. (**b**) photo of characterization test of HESM. (**c**) Charging circuit diagram of HESM for lithium-ion battery. (**d**–**f**) Voc, ISc, and Qsc of HESM. (**g**) Charging curve of a 33 μF capacitor charged by DC-EMG, PENG, and HESM. (**h**) Charging curve on various capacitances for HESM. (**i**) Charging curve of 30 mAh lithium-ion battery.

Herein, we characterized the output performance of a HESM after hybridizing the two modules. Both the DC-EMG and PENG modules utilize a servo motor to drive the watch case to rotate with a coupling, which in turn drives the swinging of the watch strap (Figure 3a,b). This motion is similar to the way a person normally grips the watch case to swing the watch strap. Figure 3c shows the circuit diagram for charging the lithium-ion battery or supplying power to the electrical device by DC-EMG and PENG modules. The DC-EMG module is connected to a boost module, which is consisted of an inductor, a chip-controlled automatic switch, a diode, and a small capacitor. The boost module converts the voltage from DC-EMG into a stable 5 V output, which is ideal for supplying power to the lithium-ion battery. Meanwhile, the PENG module is connected to the rectifier bridge and then connected to the lithium-ion battery.

The HESM was characterized according to the circuit diagram of Figure S8. The positive electrode of the DC-EMG was connected to a diode, and the PENG passed through a rectifier bridge, then the two modules were connected in parallel. The open-circuit voltage and short-circuit current of HESM were approximately 69 V and 87 mA, respectively, with an average transferred charge of 10.5 mC per half cycle of motion (Figure 3d–f). It exhibited the traits of high voltage of a PENG and high current of an EMG, and marginally enhances the output of a DC-EMG and PENG alone. We compared the charging curves of a DC-EMG, PENG, and HESM for a 33 μF capacitor and found that the HESM had better charging performance (Figure 3g). During the first few seconds of charging, the DC-EMG module was mainly responsible for increasing the capacitor voltage, which quickly rose to around 3.7 V. However, since the maximum output voltage of the DC-EMG was around 3.7 V, the voltage could not be increased any further and the charging curve became stable. On the other hand, although the charging speed of the PENG module was slower, it could continuously charge the capacitor to a higher voltage. The HESM combined the advantages of these two modules, utilizing the DC-EMG to rapidly increase the capacitor voltage to 3.6 V, after which the PENG dominated the charge effect, and charged the capacitor to 9.6 V at 200 s. We also characterized the charging performance for a 1 F capacitor (Figure S9). After about 500 s, the DC-EMG charged the capacitor to 1.28 V, the PENG charged it to 0.08 V, and the HESM charged it to 1.78 V. We further characterized the charging performance of the HESM for capacitors of different capacitance and found that the HESM could charge a 1.5 F capacitor to 1.14 V in 200 s (Figure 3h). Finally, we employed the circuit depicted in Figure 3c to charge a 30 mAh commercial lithium-ion battery. (Figure 3i). The depleted battery could be charged from 2.35 V to 3.35 V in 1000 s, demonstrating the charging capability of the HESM for emergency power supply.

### 3.4. Principle and Characterization of CPMS

The electric signal generated by a CPMS is based on the coupling of the TENG and PENG principles, with the electrical output of CPMS being the forward superposition of the TENG and PENG outputs. TENGs and PENGs are both methods of converting mechanical energy into electrical energy. A TENG utilizes the frictional electrification of materials, and relies on the variation of distance between electrodes and dielectric materials to produce a capacitance change, resulting in the generation of an electric potential difference due to electrostatic induction, while a PENG utilizes the change in electric dipole moment caused by the structural deformation of asymmetric crystals under external stress, resulting in the generation of opposite charges on the surface of piezoelectric materials and the formation of a potential difference.

**Figure 4:**
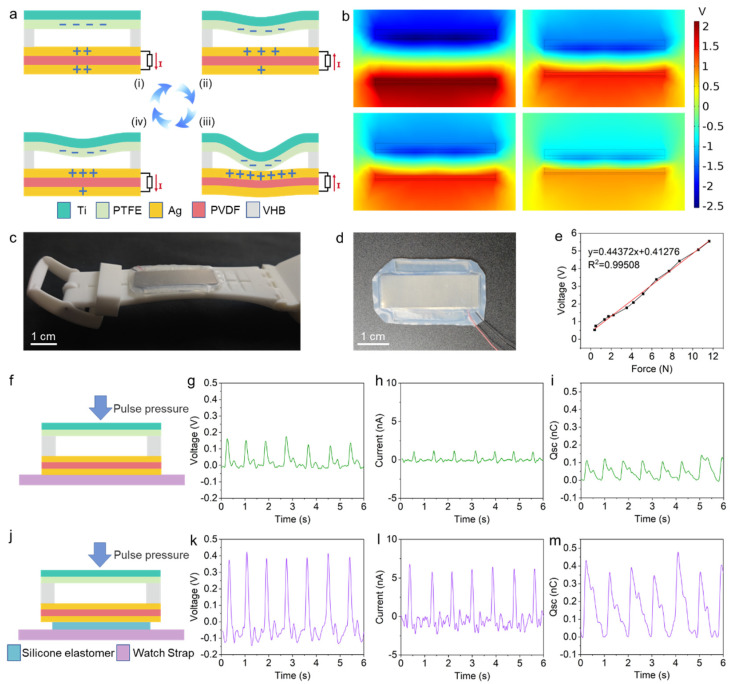
Characterization of CPMS. (**a**) Working principle of the pulse sensor. (**b**) Simulation result of the induced potential differences in a full cycle. (**c**,**d**) Photos of CPMS. (**e**) Overall linearity of the applied force and the open-circuit voltage of CPMS. (**f**–**i**) Schematic diagram and Voc, Isc, and Qsc of pulse characterization test without silicone elastomer for CPMS. (**j**–**m**) Schematic diagram and Voc, Isc, and Qsc of pulse characterization test with silicone elastomer for CPMS.

The working principle of a CPMS is shown in Figure 4a. When the upper layer of the CPMS, consisting of Ti and PTFE, undergoes compression and rebound caused by a pulse wave, it generates a negative charge on the surface of PTFE. In the state shown in Figure 4a (i), the distance between PTFE and the two Ag electrode layers is much greater than the distance between the two electrode layers, so the capacitance between PTFE and the two Ag electrode layers is approximately equal. Due to electrostatic induction, in the short-circuit state, the upper and lower Ag electrode layers carry the same amount of positive charge, and the total charge of both electrodes is equal to the surface charge on PTFE.

As the pulse pressure increases (Figure 4a (ii)), the PTFE moves closer to the lower structure layer, and the capacitance between the PTFE and the upper Ag electrode layer increases dramatically. As a result, electrons from the upper silver electrode layer are transferred to the lower silver electrode layer due to the potential difference. When the pulse pressure continues to increase (Figure 4a (iii)), the PTFE makes contact with the upper Ag electrode layer and squeezes the PVDF, resulting in an infinite capacitance between the upper Ag electrode layer and the PTFE. Due to the electrostatic induction principle of the TENG, all negative charges move to the lower surface of the PVDF. Meanwhile, according to the piezoelectric principle, the potential of the lower Ag electrode layer is higher than that of the upper Ag electrode layer due to the compression of the PVDF. It causes all negative charges to move to the lower surface of the PVDF, resulting in the superposition of the TENG and PENG outputs. When the pulse pressure decreases (Figure 4a (iv)), the PTFE separates from the upper Ag electrode layer, and the potential difference on the PVDF surface gradually disappears. The capacitance between the PTFE and the upper layer Ag electrode decreases, causing a large amount of negative charge to move to the upper Ag electrode layer. When the pulse pressure returns to the initial state, the device returns to state (i).

A TENG is more sensitive to displacement changes, while a PENG is more sensitive to changes in strain or stress. The coupling of the TENG and PENG enables the CPMS to monitor changes in the direction of displacement of the pulse wave and also to monitor apparent changes in the pressure wave generated by the pulse wave. Therefore, the CPMS can realize the sensitive monitoring of pulse wave signals. The pulse wave signals contain important health information about the human body, such as arterial stiffness, heart rhythm, and cardiovascular disease risk [41]. From the result of the simulation of the electric field by COMSOL, it can be observed that when the upper and lower structure layers are far apart, the potential between the two electrode layers is approximately equal (Figure 4b). When they approach together, the potential difference between the two Ag electrode layers is maximized.

Figure 4c,d shows the CPMS and its position on the watch strap. The dark gray layer with metallic luster in the photo is the Ti metal layer, which is in contact with the skin after encapsulation. The upper structural layer is composed of a Ti sheet as a hard elastic substrate, which can transmit the pulse wave in the skin to the CPMS more sensitively and convert it into a real-time pressure wave. Then the pressure drives the contact-separation and squeeze deformation of the PTFE in the upper structural layer and the PVDF in the lower structural layer. The middle support layer is composed of four VHB double-sided adhesive tapes, with two tapes placed on each side between the upper and lower structural layers for support and connection. The other two VHB tapes are placed outside the gaps on the other two sides of the structural layer to provide more external support to the intermediate movable part between the upper and lower structural layers during encapsulation, to prevent the CPMS from being flattened and causing output reduction. Moreover, due to the increased volume of air inside, more space for deformation is left for the CPMS. In addition, the CPMS can be well fixed to the strap after encapsulation. This is because the two VHBs outside the structural layer provide more constraints to the CPMS, forming a statically indeterminate structure. A statically indeterminate structure is a mechanical structure with redundant constraints. Additional redundant constraints can bring about more generalized forces than a simple statically determinate structure. When the statically indeterminate structure is subjected to external forces of different directions and magnitudes, the redundant constraints can be adaptively deformed according to the geometric structure, material elastic modulus, and the positional relationship of the constraints, resulting in smaller deformations and displacements of the structure. Therefore, the statically indeterminate structural design allows the wearer to maintain a relative stillness between the CPMS and the watch strap during movement.

The thickness of the supporting layer and the length of the structural layer were explored and optimized. We found that the pulse signal from the CPMS was strongest when the supporting layer between the upper and lower structural layers was 0.8 mm thick (Figure S10). In addition, while keeping the width of the structural layer constant, the influence of the length of the structural layer on the pulse signal strength was explored (Figure S11). The results indicate that when the length of the structural layer is 3 cm, the pulse signal is strongest. Finally, under the impact load of 5 N, 100,000 fatigue test experiments were carried out on the CPMS, and the output voltage was kept at about 25 V, and the voltage hardly changed during the whole process. It can be seen that the CPMS has high stability (Figure S12).

The CPMS exhibits a good force–voltage linear relationship within a relatively large force range (Figure 4e). After encapsulation, when the CPMS is directly bonded to the strap without using silicone elastomer, the open-circuit voltage, short-circuit current, and short-circuit transfer charge obtained during pulse monitoring while the wearer is sitting still are 0.15 V, 1.17 nA, and 0.11 nC, respectively (Figure 4g–i). When the CPMS is bonded to the strap using silicone elastomer, the open-circuit voltage, short-circuit current, and transferred charge are increased to 0.38 V, 6.44 nA, and 0.40 nC, respectively. The electrical output is greatly improved because the silicone elastomer can fill the natural arches and minor wrinkles between the CPMS and the watch strap, reducing the pre-tension of adhesion. When the pulse wave drives the motion of the CPMS, there is more deformation space available for the PVDF of the lower structural layer. In this way, the CPMS can generate greater strain and displacement under the same pulse pressure. Therefore, the introduction of silicone elastomer can significantly improve the pulse signal of the CPMS.

### 3.5. Demonstration of the Applications of SPMB

**Figure 5:**
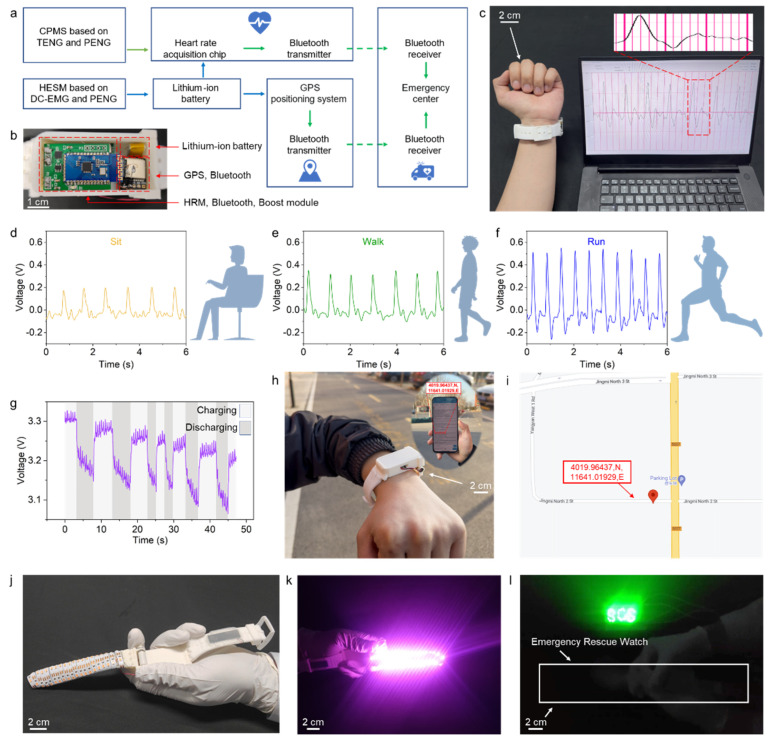
Applications of SPMB. (**a**) Flow diagram of personal rescue by SPMB. (**b**) Photo of functional electronic component in watch case. (**c**) Photo of pulse monitoring. (**d**–**f**) Pulse monitoring in different motion states. (**g**) Charge curve of a 30 mAh lithium-ion battery by SPMB and discharge by GPS and Bluetooth. (**h**,**i**) Photo and electronic map of GPS positioning by functional electronic component. (**j**,**k**) Light strip lit by SPMB. (**l**) SOS signal light lit by SPMB.

The proposed SPMB consists of the CPMS, HESM, and functional electronic components, enabling the real-time wireless transmission of pulse signal and position information, and illumination of the distress signal light in emergencies. Figure 5a shows the flow diagram of the SPMB for personal rescue. The HESM collects energy and charges the lithium-ion battery for storage. The lithium battery provides power to the HRM module, GPS positioning module, and Bluetooth module. The HRM processes the pulse wave signal collected by the CPMS, and sends it to the mobile phone by Bluetooth. Meanwhile, the GPS positioning module receives the latitude and longitude information and sends them to the mobile phone via another Bluetooth module. In the future, through integration with the commercial smartwatch, it is possible that, when the wearer’s wave changes dramatically, the mobile phone will send the pulse and location information to request help. Figure 5b shows the photo of the functional electronic elements inside the watch case. Figure 5c shows the real-time monitoring of the pulse wave signal by the SPMB. The pulse signals were wirelessly transmitted via the Bluetooth module inside the watch case to a computer for display. The stable pulse information was demonstrated when the wearer’s arm is still, swinging slowly, and swinging rapidly (Supplementary Video S1). Figure 5d–f shows the pulse signals collected by the SPMB when the wearer is in a static, walking, and running state, respectively. The SPMB can achieve stable pulse monitoring in different motion states, demonstrating anti-interference and stability.

Figure 5g shows the charging and discharging curve of a 30 mAh lithium-ion battery by the SPMB while simultaneously driving the GPS module and Bluetooth module. During a cold start, the GPS module is in its maximum power consumption state. Figure 5h–i show the latitude and longitude information obtained by the GPS module in the watch case, which is transmitted to the mobile phone for display through the Bluetooth module. In addition, the SPMB can light up the light strip and rescue light when the watch strap is swung (Supplementary Videos S2 and S3), achieving illumination and distress signal flashing.

## 4. Conclusions

In summary, a self-powered multifunctional bracelet (SPMB), integrated with an EMG-PENG hybrid energy supply module (HESM) and a TENG-PENG coupled pulse monitoring sensor (CPMS), is proposed for long-term physiological monitoring and personal emergency rescue for outdoor workers and explorers. Based on the inherent structural design of the watch, a DC-EMG is used to replace the hinge structure at the connection between the watch case and watch strap, and a PENG module is encapsulated in the watch strap. Through structure and parameter optimization, the HESM can well match the frequency and manner of watch strap swing. By simultaneously collecting the rotational kinetic energy from the watch strap swinging and the elastic potential energy from the watch strap deformation, the HESM can generate a voltage of 69 V and a current of 87 mA, and charge a commercial lithium-ion battery of 30 mAh from 2.35 V to 3.35 V within 1000 s. The coupling of a TENG and PENG can sensitively detect physiological signals of pulse wave. Utilizing a statically indeterminate structure design, and employing silicone elastomer to combine the CPMS with a watch strap to reduce pre-tension, the CPMS can stably monitor pulse signals during movement, with strong anti-interference ability. With the assistance of functional electronic components in the watch case and the emergency power supply of the HESM, the pulse wave signal and position information of the wearer can be transmitted through the SPMB wirelessly. By flipping the watch strap slightly with the hand, the SPMB can directly drive the rescue light and realize the lighting function, showing the potential for personal rescue in emergencies. In addition, the universal compact design allows the SPMB to be easily integrated with mechanical or electronic watches on the market, with wide application prospects.

## Data Availability

The data presented in this study are available on request from the corresponding author.

## Supplementary Materials

The following supporting information can be downloaded at: https://www.mdpi.com/article/10.3390/bios13050552/s1, Figure S1: Working principle of the overrunning clutch; Figure S2: Magnetic flux density over a period simulated by COMSOL; Figure S3: Voltage curve of DC-EMG module by electromagnetic simulation; Figure S4: The voltage curve of piezoelectric material during one cycle simulated by COMSOL; Figure S5: Voltage curve of PENG module by COMSOL simulation; Figure S6: Durability test of DC-EMG; Figure S7: Durability test of PENG; Figure S8: Circuit diagram of HESM directly driving load or charging a capacitor; Figure S9: Charging curve of a 1F capacitor charged by DC-EMG, PENG, HESM; Figure S10: Effect of the support layer thickness of CPMS on pulse signal; Figure S11: Effect of structural layer length of CPMS on pulse signal; Figure S12: Durability test of CPMS; Video S1: Pulse monitoring during static and dynamic of the arm using SPMB; Video S2: Powering light strip by SPMB; Video S3: Powering rescue light by SPMB.
