# Supplementary material for: A Self-Powered Multifunctional Bracelet for Pulse Monitoring and Personal Rescue
Source: Biosensors (Basel). 2023 May 16;13(5):552. doi: 10.3390/bios13050552 (PMC10216715; doi:10.3390/bios13050552)
Supplement: Supplementary file 1 [file biosensors-13-00552-s001.zip › biosensors-2349365-supplementary.pdf]

biosensors

Supporting information

A Self-Powered Multifunctional Bracelet for Pulse Monitoring and Personal Rescue

Wei Sun [1,2,†], Jiangtao Xue [1,3,†], Puchuan Tan [1,2], Bojing Shi [4], Yang Zou [1,3,*], Zhou Li [1,2,5,*]

1 Beijing Institute of Nanoenergy and Nanosystems, Chinese Academy of Sciences, Beijing 101400, China; sunwei@binn.cas.cn (W.S.); xuejiangtao@binn.cas.cn (J.X.); tanpuchuan@binn.cas.cn (P.T.);
2 School of Nanoscience and Engineering, University of Chinese Academy of Sciences, Beijing 100049, China;
3 School of Life Science, Institute of Engineering Medicine, Beijing Institute of Technology, Beijing 100081, China;
4 Key Laboratory for Biomechanics and Mechanobiology of Ministry of Education, Beijing Advanced Innovation Centre for Biomedical Engineering, School of Biological Science and Medical Engineering, School of Engineering Medicine, Beihang University, Beijing 100191, China; bjshi@buaa.edu.cn (B.S.)
5 Center on Nanoenergy Research, School of Physical Science and Technology, Guangxi University, Nanning 530004, China;
* Correspondence: zouyang@binn.cas.cn (Y.Z.); zli@binn.cas.cn (Z.L.);
† These authors contributed equally to this work.

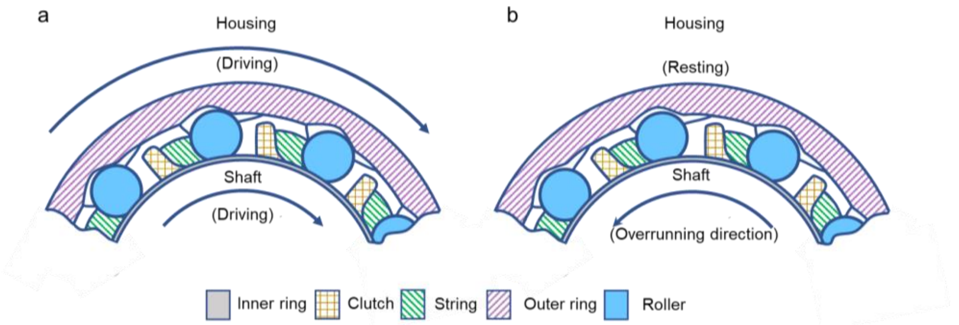

Figure S1. Working principle of the overrunning clutch. (**a**) Clutch in engagement. (**b**) Clutch overrun.

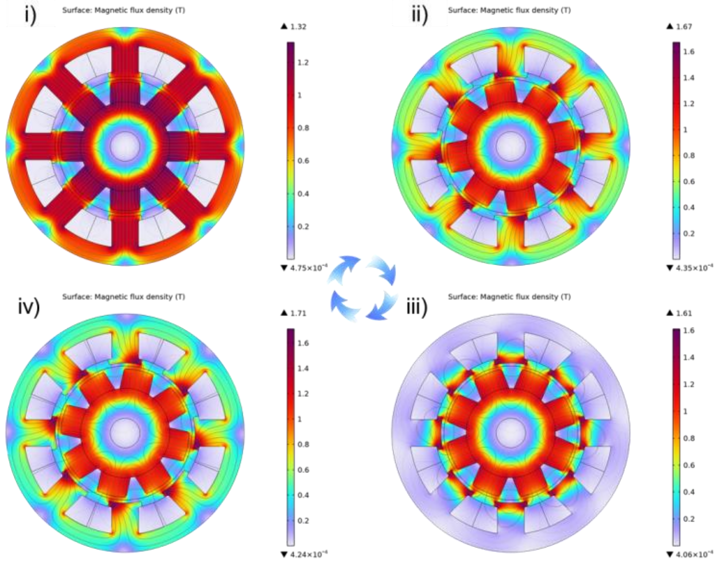

Figure S2. Magnetic flux density over a period simulated by COMSOL.

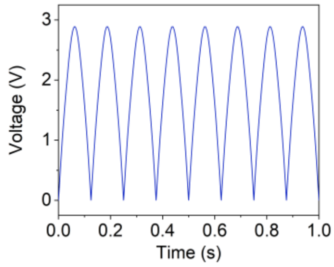

Figure S3. Voltage curve of DC-EMG module by electromagnetic simulation.

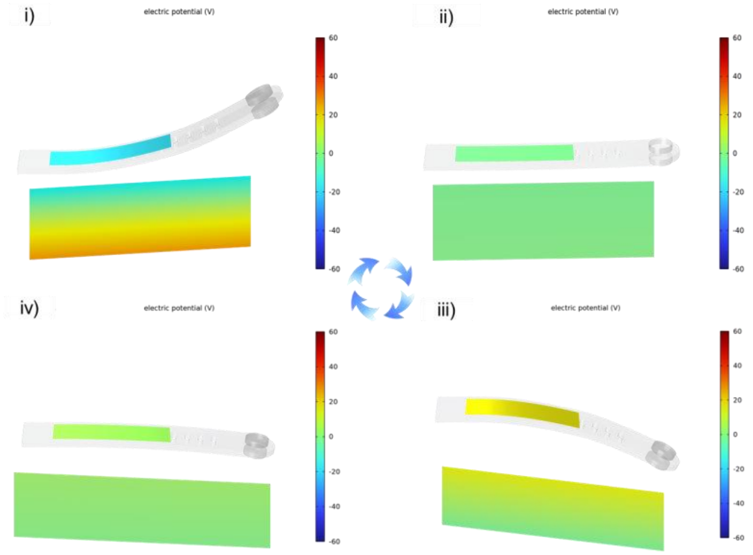

Figure S4. The voltage curve of piezoelectric material during one cycle simulated by COMSOL.

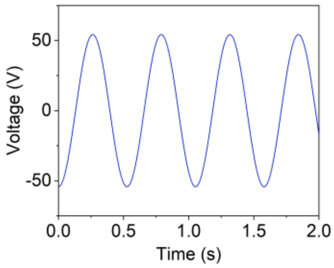

Figure S5. Voltage curve of PENG module by COMSOL simulation.

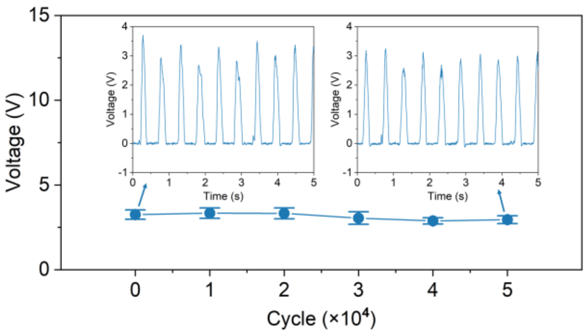

Figure S6. Durability test of DC-EMG.

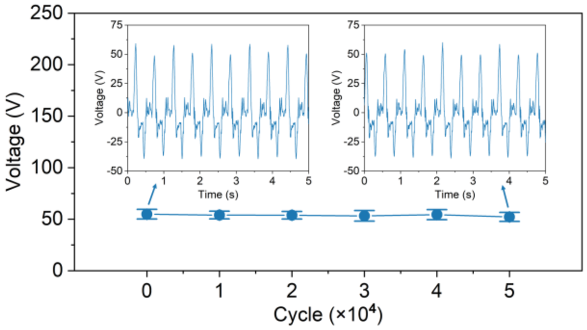

Figure S7. Durability test of PENG.

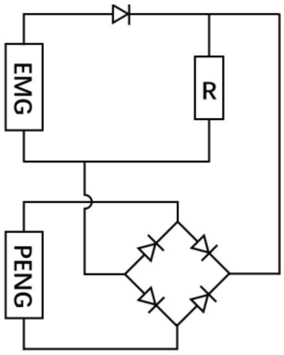

Figure S8. Circuit diagram of HESM directly driving load or charging a capacitor.

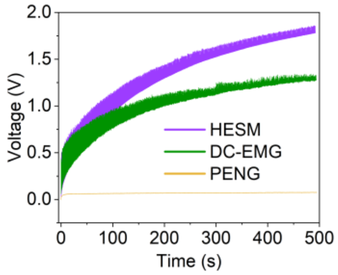

Figure S9. Charging curve of a 1F capacitor charged by DC-TENG, PENG, HESM.

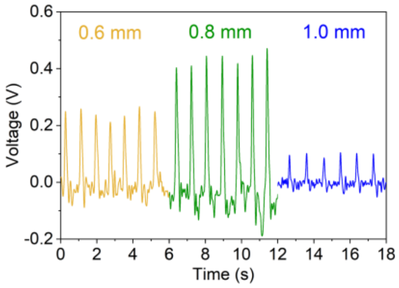

Figure S10. Effect of the support layer thickness of CPMS on pulse signal.

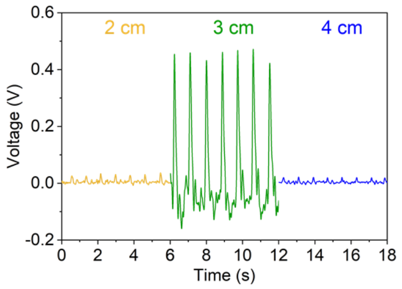

Figure S11. Effect of structural layer length of CPMS on pulse signal.

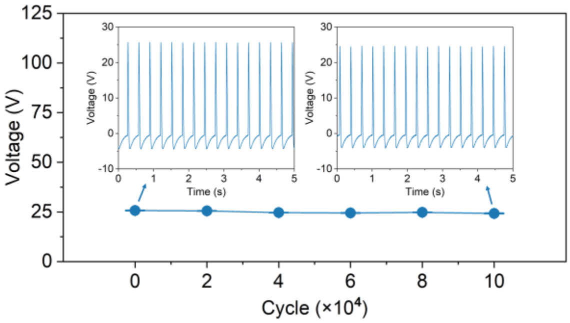

Figure S12. Durability test of CPMS.
